# A synoptic literature review of animal models for investigating the biomechanics of knee osteoarthritis

**DOI:** 10.3389/fbioe.2024.1408015

**Published:** 2024-07-25

**Authors:** Luyang Xu, Zepur Kazezian, Andrew A. Pitsillides, Anthony M. J. Bull

**Affiliations:** ^1^ Department of Bioengineering, Imperial College London, London, United Kingdom; ^2^ Centre for Blast Injury Studies, Imperial College London, London, United Kingdom; ^3^ Skeletal Biology Group, Comparative Biomedical Sciences, Royal Veterinary College, London, United Kingdom

**Keywords:** osteoarthritis, preclinical models of osteoarthritis, biomechanical factors, biomechanically induced models, biomechanical measurement techniques

## Abstract

Osteoarthritis (OA) is a common chronic disease largely driven by mechanical factors, causing significant health and economic burdens worldwide. Early detection is challenging, making animal models a key tool for studying its onset and mechanically-relevant pathogenesis. This review evaluate current use of preclinical *in vivo* models and progressive measurement techniques for analysing biomechanical factors in the specific context of the clinical OA phenotypes. It categorizes preclinical *in vivo* models into naturally occurring, genetically modified, chemically-induced, surgically-induced, and non-invasive types, linking each to clinical phenotypes like chronic pain, inflammation, and mechanical overload. Specifically, we discriminate between mechanical and biological factors, give a new explanation of the mechanical overload OA phenotype and propose that it should be further subcategorized into two subtypes, post-traumatic and chronic overloading OA. This review then summarises the representative models and tools in biomechanical studies of OA. We highlight and identify how to develop a mechanical model without inflammatory sequelae and how to induce OA without significant experimental trauma and so enable the detection of changes indicative of early-stage OA in the absence of such sequelae. We propose that the most popular post-traumatic OA biomechanical models are not representative of all types of mechanical overloading OA and, in particular, identify a deficiency of current rodent models to represent the chronic overloading OA phenotype without requiring intraarticular surgery. We therefore pinpoint well standardized and reproducible chronic overloading models that are being developed to enable the study of early OA changes in non-trauma related, slowly-progressive OA. In particular, non-invasive models (repetitive small compression loading model and exercise model) and an extra-articular surgical model (osteotomy) are attractive ways to present the chronic natural course of primary OA. Use of these models and quantitative mechanical behaviour tools such as gait analysis and non-invasive imaging techniques show great promise in understanding the mechanical aspects of the onset and progression of OA in the context of chronic knee joint overloading. Further development of these models and the advanced characterisation tools will enable better replication of the human chronic overloading OA phenotype and thus facilitate mechanically-driven clinical questions to be answered.

## 1 Introduction

Osteoarthritis (OA) is a highly prevalent chronic disease that affects the joints and contributes significantly to a loss of physical function (Kloppenburg and Berenbaum, 2020). It is characterised by pain, sometimes linked to cartilage disintegration, bone remodelling and inflammation of the synovium. In the United Kingdom, the incidence of any type of OA in 2017 was 10.7%, with a 1-year consistent frequency of 6.8/10^3^ adults (≥20 years) and 40.5/10^3^ adults (≥45 years) (Swain et al., 2020; Allen et al., 2022). Pain associated disability as a result of OA represents a significant burden (22% of total ill health burden) in societal and personal costs (Versus Arthritis, 2019; Wolf et al., 2019). Internationally, hip and knee OA is the 11th highest cause of disability (GBD, 2017 Disease and Injury Incidence and Prevalence Collaborators, 2018). Knee OA (KOA) is the most prevalent type of OA and is increasing in incidence as obesity and life span increase (Wallace et al., 2017; Hunter and Bierma-Zeinstra, 2019). The latest musculoskeletal calculator (published in 2019) estimated that in 2012 18.2% of the English population over 45 years old had clinically diagnosed KOA, 1/3 of which was severe (Versus Arthritis, 2019).

KOA is historically categorised into two main types, primary OA- also known as idiopathic OA- which is characterised by natural cartilage degeneration during ageing as a result of unidentified reasons, and secondary OA which occurs as a result of known medical conditions or risk factors (Altman et al., 1986). However, with extensive research over the past 2 decades dedicated to understanding risk factors and aetiologies, primary OA has been further divided into genetic, aging and hormonal (estrogen-deficiency) subsets (Herrero-Beaumont et al., 2009). Secondary OA is classified into subsets like metabolic disorders, anatomical abnormalities, traumatic injury and inflammation (Arden and Nevitt, 2006).

**FIGURE 1:**
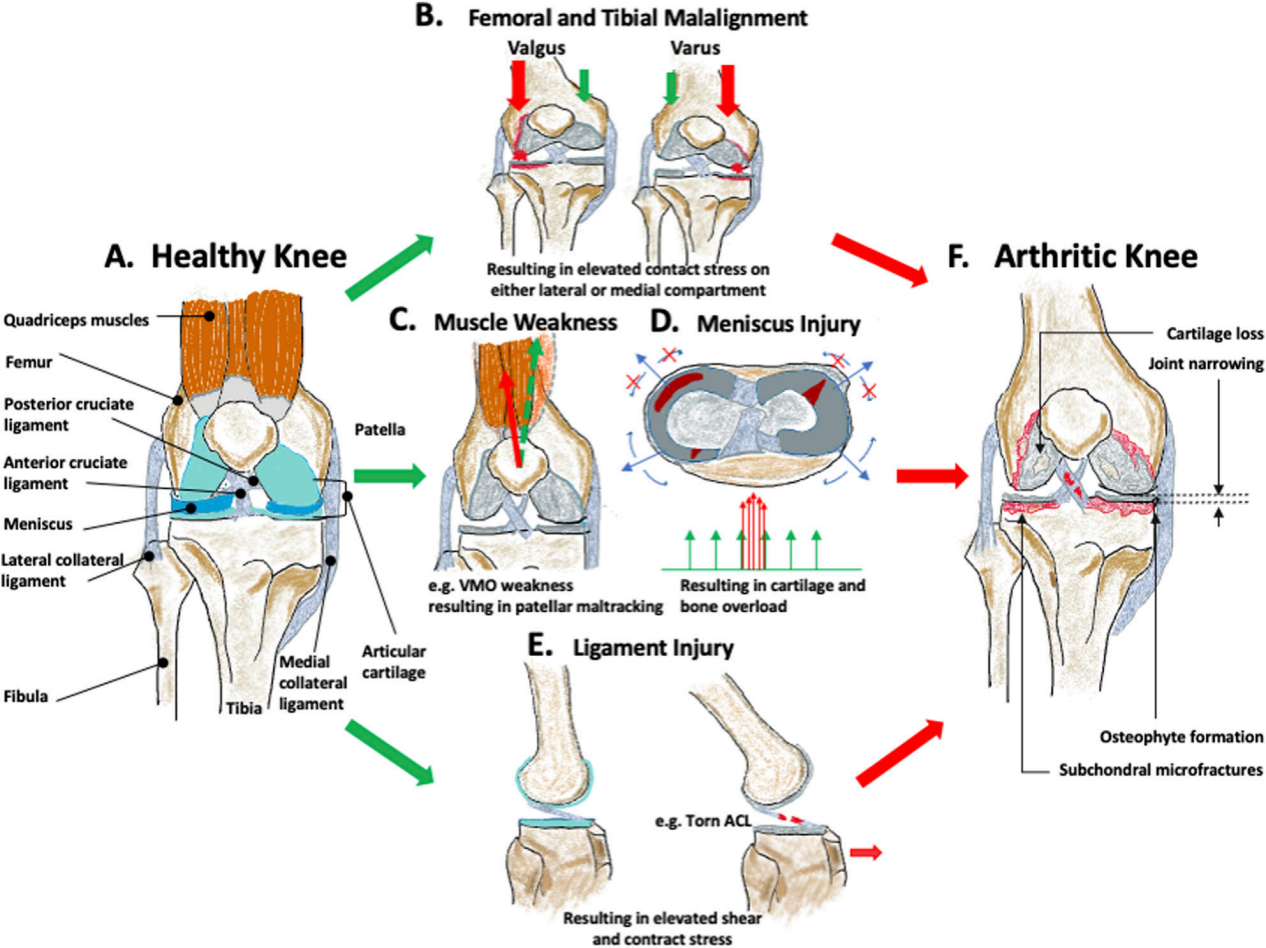
Biomechanical factors leading to osteoarthritis (OA). **(A)** represents the healthy knee. **(B)** femoral and tibial malalignment with resulting varus or valgus angulation and medial or lateral joint overload, **(C)** quadriceps muscular weakness leading to muscle imbalance and local joint overload, **(D)** meniscus injury leading to increased contact stress, **(E)** ligament injury leading to changes in the overall knee mechanics. All of these will eventually contribute to increasing the mechanical stress on the healthy knee and induce joint structural changes, including cartilage loss, joint narrowing, osteophyte formation, and subchondral microfractures resulting in, **(F)** arthritic knee.

Beyond these classic subsets centred on aetiological risk, recent studies propose use of clinical phenotype and endotype concepts to categorize this heterogeneous disease (Deveza and Loeser, 2018; Deveza et al., 2019; Mobasheri et al., 2019); a clinical phenotype refers to observable characteristics of an individual resulting from the interplay between genotype and environment (Mobasheri et al., 2019). Dell’Isola *et al* classified knee OA into six clinical phenotypes based on a qualitative meta-analysis of existing data from 24 studies: 1) long-lasting pain; 2) upregulation of inflammatory markers; 3) metabolic disorder (estrogen imbalance, dyslipidemia, diabetes, and obesity); 4) changes in bone and cartilage homeostasis; 5) altered joint mechanics (medial meniscus overload or varus malalignment) (Figure 1B); and 6) minor joint disease (slight clinical symptoms with chronic progression) (DellIsola et al., 2016). The complexity is confounded by these phenotypes not necessarily being discrete, with multiple phenotypes potentially co-existing (Bierma-Zeinstra and van Middelkoop, 2017). As a result, reaching a consensus on diagnosing each specific subgroup is challenging due to phenotype overlaps, meaning no specific biomarkers or tests can be uniquely applied. OA endotypes (or mechanistic phenotypes) are defined by distinct pathobiological molecular mechanisms and signaling pathways, making them identifiable by specific biomarkers (Anderson, 2008). This distinction is valuable for targeted anti-cytokine therapy. Inflammation, metabolism, cell senescence, and bone and cartilage endotypes are considered constituent molecular endotypes of each phenotype (Mobasheri et al., 2019).

KOA (Figure 1A) is insidious and is defined as the degeneration of the articular cartilage in the weight-bearing region, with structural changes of the surrounding bone, and joint inflammation, irrespective of the presence of clinical symptoms (Kraus et al., 2015; Lespasio et al., 2017) (Figure 1F). This joint degeneration is affected by a combination of mechanical and biological effects (Sandell and Aigner, 2001). Epidemiologically, OA risk factors can be categorised into *individual-centred* (or systemic) factors such as age, sex, race, genetic background, metabolic disorder, endocrine, diet, bone density, and *joint-related* factors such as different shapes of the articulating surfaces, muscle weakness, occupation/sports, and injury and joint overloading (Vina and Kwoh, 2018). Biomechanical factors are the predominant theme associated with these joint-related factors, and this is the focus of much recent KOA research (Glyn-Jones et al., 2015; van Tunen et al., 2016). There is also increasing evidence that OA onset is mechanically driven and the onset is strongly correlated with altered mechanical properties of subchondral bone (Day et al., 2001; Hayami et al., 2006).

Biomechanical factors play an important role in understanding the pathogenic mechanism of all forms of KOA and in helping to address clinical questions. For example, it is reported that frontal plane knee malalignment has the strongest relation with KOA development (Palazzo et al., 2016). The natural knee alignment is around 1° varus (Cooke et al., 2007) and the ideal realignment angle ranges from 1° to 13° valgus (Dugdale et al., 1992; Cooke et al., 2007; Tsukada and Wakui, 2017; Sethi et al., 2020). However, these data are all derived from retrospective studies, which are population-based and laboratory experiments to confirm these findings are lacking. Moreover, despite OA (Figure 1) being a progressive disease, symptomatic OA is usually only diagnosed in patients with clinical symptoms and imaging evidence at an advanced stage (Hayami et al., 2006), which is likely irreversible. Therefore, it is imperative to devote research efforts to detect biomechanical mechanisms at the initial and early stages of OA. This is key not only for studying disease aetiology, but would also represent a big step towards developing preventative treatment and in diagnosing pre-radiographic OA.

Experiments to investigate these biomechanical factors are difficult in human studies as the mechanical intervention and direct outcomes are hard to perform and observe independently (Wendler and Wehling, 2010), and the available human tissue diagnosed as OA is not in the early stage (Hayami et al., 2006). *Ex vivo* joint models cannot truly mimic musculoskeletal mechanics and the arthritic environment. Thus, *in vivo* experimental animal models are important in studying the onset and early stages of this disease, particularly when investigating biomechanical aspects (Kuyinu et al., 2016). The two main goals of using OA *in vivo* models are: 1) to understand the pathophysiology of the disease, especially in the early stages, and 2) to test the safety and efficacy of different therapeutics developed before entering into clinical trials (Kuyinu et al., 2016). Recent advances in measurement techniques for *in vivo* animal studies have enabled and improved the analysis of OA and reduced animal usage (Teeple et al., 2013). In the light of recent work on *in vivo* animal models and methodological advances on biomechanical aspects of KOA, the purpose herein is to conduct a narrative review on the use of preclinical *in vivo* models, and advanced measurement techniques focusing specifically on biomechanical aspects in the context of the identified human clinical sub-division of OA.

## 2 Biomechanical factors in OA

Biomechanical factors are defined herein as joint-related, OA-linked, features based on epidemiological statistics. These are subdivided into either those anatomical relationships influencing the joint, or those that are the consequence of functional joint usage (Glyn-Jones et al., 2015; van Tunen et al., 2016). The following section presents current evidence on biomechanical-related risk factors associated with OA.

Anatomical factors include those derived from individual joint morphology and limb alignment (Glyn-Jones et al., 2015), where these can be variations in the shape of the tibia, femur, or patella. The anatomy is the product of genetics and adaptation to use, and the reasons for the adaptive changes are not fully known, but are likely to align with Wolff’s law, which describes how bone tissue can be remodelled in response to prevailing mechanical environment (Chen et al., 2010). These morphological variations can, therefore, be created by habitual joint loading experiences, but will also then induce corresponding shifts in joint loading and so may initiate or accelerate OA (Neogi et al., 2013). The morphological variation can predict the onset of OA, a year earlier than visible radiographic changes (Neogi et al., 2013). Extensive variation, recognized as knee malalignment (i.e., varus or valgus alignment) is also a strong risk factor in OA onset and progression (Tanamas et al., 2009). It is defined as a shift from the collinear mechanical alignment of hip, knee and ankle to either a medialised (varus) or lateralised (valgus) loading distribution (Sharma et al., 2001). The alignment changes can be a result of localised morphological deviation. In the varus knee, the mechanical axis (from mid femoral head to mid ankle) will pass medial to the centre of the knee. This induces an adduction moment that increases the force on the medial tibiofemoral compartment; the opposite happens in the valgus knee (Tetsworth and Paley, 1994). This loading imbalance contributes to OA progression (Sharma et al., 2001), with a 1° varus deviation out of the natural physiological range of alignment, disproportionally increasing the medial load by 5% (Schipplein and Andriacchi, 1991; Halder et al., 2012), As such, varus and valgus malalignment (Figure 1B) is a known independent risk factor in the progression of medial and lateral OA (Tanamas et al., 2009). Of these, varus alignment is more common in the knee OA population and is significantly linked with OA development (Brouwer et al., 2007).

Functional factors that are a consequence of challenging joint usage include previous knee injury, poor muscle function, reduced proprioceptive acuity, activities (occupational activity and sports activity), and joint laxity (Bennell et al., 2012; Richmond et al., 2013; Glyn-Jones et al., 2015; van Tunen et al., 2016). Knee injury mainly includes knee ligament ruptures, sprains or meniscal tears (Thomas et al., 2017), which carry a high risk of post-traumatic OA (PTOA) onset compared to uninjured individuals (Thomas et al., 2017). There is some evidence of the high OA incidence in athletes being mainly due to joint injury (Bennell et al., 2012). Meniscal and anterior cruciate ligament (ACL) injuries each account for one-quarter of all knee injuries (Thomas et al., 2017). Ligament injuries can cause changes in joint mechanics, including translation and shear, leading to overload (Figure 1E) (Kernozek et al., 2013). Simply, meniscal tears decrease the contact area between cartilaginous surfaces thus increasing contact stresses during load transfer (Figure 1D) (Masouros et al., 2008). Muscle weakness (Figure 1C), including athrogenic muscle inhibition, was previously thought to be a secondary factor that results from progression of OA damage, but recent population-based evidence shows that muscle dysfunction can be a primary risk of OA initiation, preceding knee pain and muscle atrophy (Slemenda et al., 1997). Quadriceps weakness specifically is a better predictor of joint pain and joint space narrowing than radiographic evidence (Palmieri-Smith et al., 2010).

Impaired knee proprioception refers to a deteriorated accuracy in position and motion sense, caused by impaired articular mechanoreceptors (Knoop et al., 2011). Reduced proprioceptive accuracy has been found in OA patients and this causes a decrease in knee stabilization and joint movement coordination, both of which are directly related to mechanics, and has a significant association with knee pain (Knoop et al., 2011; van Tunen et al., 2018). These factors are linked, as the loss of muscle strength can be attributed to afferent sensory dysfunction (i.e., impaired proprioception) and the subsequent reduction in efferent neuron stimulation (Slemenda et al., 1997). Although there is currently no robust evidence that dysfunctional proprioception causes OA onset, it has been proposed as a putative OA risk factor (Roos et al., 2011). Activities (e.g., physical, occupational and sporting) are controversial factors for knee OA, as cartilage homeostasis is known to rely upon moderate loading but could change to catabolic metabolism in high intensity and long duration, abnormal mechanics (Griffin and Guilak, 2005). Heavy occupational lifting, frequent deep knee flexion (squatting, kneeling) and sports activity (at elite level) with significant impact, twisting, turning and running have a demonstrated higher OA risk (Richmond et al., 2013). In contrast, there is no evidence that physical activity (moderate sports) and recreational sports activity, without suffering injury, increase the risk of OA (Bennell et al., 2012; Richmond et al., 2013). Some large studies have found that ice hockey and soccer players, wrestlers and weightlifters have a much higher incidence of OA than endurance sport athletes (Kujala et al., 1994; Tveit et al., 2012). This aligns with the notion that there is a biomechanical tolerance level in weight-bearing joints; this level is not currently known (Thelin et al., 2006). However, there could simply be a higher incidence of injury in those sports in which higher biomechanical loads are engendered.

Joint laxity–loss of stability with deficiencies in the primary soft-tissue stabilisers such as the cruciate ligaments, secondary stabilisers such as the capsule, or in alignment–is another biomechanical-related OA risk factor (van Tunen et al., 2016). Evidence has shown that patients with medial knee OA always have laxity, usually in the varus-valgus direction (van Tunen et al., 2018). All of these factors are related to biomechanics (loading, motion) and in general, alterations in these factors will change knee joint loading (van Tunen et al., 2016). Acute or long-term overuse changes in loading will subsequently induce the structural, biological and mechanical level changes and present as clinical symptoms.

Although biomechanical risks can be independent, direct-causative factors in some cases of knee OA, all OA phenotypes have a mechanical component, and all risk factors can induce shifts in mechanical stress in chondrocyte pericellular matrix (Brandt et al., 2009; Zhao et al., 2020). For example, obesity is an important OA risk factor; not only because the systemic inflammation arising from adipokines can promote local joint inflammation (Berenbaum et al., 2013; Robinson et al., 2016), but because obesity can also interact with confounding biomechanical factors, evidenced by less frequent cyclic physiologic joint loading (lowering risk) and altered gait and increased peak knee force (which both increase risk) (Aaboe et al., 2011; Issa and Griffin, 2012). At the molecular and cellular level, cartilage degradation is generally affected by biological and mechanical stimuli (Sandell and Aigner, 2001) and many risk factors induce OA by combined inflammation and stress-induced mechanotransduction signaling pathways in chondrocytes (Guilak, 2011). Recent advances in our grasp of chondrocyte mechano-signaling make the mechanism by which biomechanical factors may contribute to OA clearer. It is now evident that the pericellular matrix of the cartilage chondrocyte transmits the physiological mechanical stimulus (including abnormal stress, compression, fluid pressure) to cell surface mechanoreceptors (ion channels, integrins) which convert these outside physical signals to intracellular signals, to regulate a series of downstream pathways (Guilak, 2011; Gilbert and Blain, 2018; Zhao et al., 2020). This suggests that this biomechanical stress may be critical in the initiation and progression of OA, independently of the influence of inflammation. Therefore, although there is tantalising information, there is still no consensus in the understanding of the biomechanical factors underlying OA initiation and progression (Guilak, 2011; Saxby and Lloyd, 2017; Hunt et al., 2020). Some experts propose that any efforts to subdivide OA should largely consider the abnormal mechanical stress loading on the joint, and advise subclassifying OA based on mechanical abnormalities (Radin et al., 1991; Deveza et al., 2019). This highlights the need to accurately define the various mechanical factors, and the abnormal mechanical stress, contributing to the different phenotypes of OA. To identify these factors and to address this fundamental gap of mechanical stress mechanism, preclinical models should be developed that are clinically representative of biomechanically induced OA (McCoy, 2015; Kuyinu et al., 2016; Serra and Soler, 2019).

## 3 Classifying preclinical *in vivo* models of OA

As OA is a multifactorial disease driven by genetic, biological, and biomechanical factors (Glyn-Jones et al., 2015), there are several different *in vivo* models that provide a means to study its various distinct features. Each model replicates a unique OA aetiology and pathogenesis that reflects the specific mechanism of interest as well as the target uses for drug treatment (Cope et al., 2019). Generally, small mammals (nearly always rodents), including mice, rats, rabbits and guinea pigs, are considered for primary basic research (Serra and Soler, 2019), such as the study of OA pathology/pathophysiology, and assessing the effectiveness of different therapeutics (Kuyinu et al., 2016). Larger animals (dog, horse, goat, cattle) develop the disease more slowly, are more expensive, less reproducible and harder to handle (Serra and Soler, 2019), yet can be more relevant to disease heterogeneity and the anatomy, dimensions and biomechanics of human joints (Lampropoulou-Adamidou et al., 2014; Meeson et al., 2019b). Therefore, they are more widely used in translational preclinical studies, where the clinical progression of the disease and treatment are being considered.

These *in vivo* OA models are characteristically classified as: *spontaneous,* which include naturally occurring for uncertain/unknown reasons and genetically modified models, and *induced* models which can be provoked by chemical or surgical intervention (Table 1). Recently, some non-invasive induced models have been developed as alternatives to surgical models (Christiansen et al., 2015; Poulet, 2016). These OA models are typically categorised in terms of classical primary and secondary OA types. Spontaneous models characterise to a certain extent the natural progression of human primary OA with or without genetic modification. Accordingly, induced models are developed as a result of replicating specific known risk factors of secondary OA and these models are usually surgically induced, and sometimes less appropriately chemically induced (Vincent et al., 2012; McCoy, 2015). The induced methods can stimulate acute inflammation and/or alter the joint mechanics (Serra and Soler, 2019).

**TABLE 1:** Preclinical *in vivo* OA models.

OA models	Subcategory	Species	Model	Characteristics	Ref.
Spontaneous	Natural	Guinea pig Rabbit Horse Mouse Dog	Wear and tear	1. Represents human primary OA 2. Study early pathogenesis 3. Longer progression time. Small animals have shorter time required for maturity	Jimenez et al. (1997), Mason et al. (2001), McDougall et al. (2010), Arzi et al. (2012), McIlwraith et al. (2012), Staines et al. (2017b), Meeson et al. (2019a), Brioschi et al. (2020)
	Genetic	Mouse Rat	Genetic inclination (genes involved in cartilage degradation, inflammation, apoptosis and bone metabolism)	1. Study role of specific genes in OA pathogenesis 2. Help to establish molecular basis of OA. Low clinical trial translatability	Little and Zaki (2012), Miller et al. (2013), Poulet et al. (2013)
Chemical		Mouse Rat Rabbit Goat	Intra-articular injection of chemical to induce cell death or inflammation	1. Mainly inflammation model and most commonly used model in drug development 2. Used to study drugs/response to pain and inflammation 3. Simple and repeatable 4. Although produces rapid and severe joint degeneration, does not reproduce pathophysiological mechanisms	Laurent et al. (2003), Rodrigues-Neto et al. (2016), Sukur et al. (2016), Adeyemi and Olayaki (2017), GBD, 2017 Disease and Injury Incidence and Prevalence Collaborators (2018), Cheng et al. (2019), Lee et al. (2020)
Surgical	Anterior cruciate ligament transection (ACLT)	Mouse Rat Goat Rabbit	ACL injury produced by arthrotomy (medial or lateral) or arthroscopy (destabilization)	1. Most often used surgical model, suitable for pharmacologic studies 2. Leads to cartilage degradation but develops OA lesion slowly 3. Can be combined with meniscectomy to achieve advanced lesions	Jackson et al. (1993), Kamekura et al. (2005), Hayami et al. (2006), Piskin et al. (2007), Ramme et al. (2018)
	Meniscal injury	Mouse Rat Canine Goat	Partial/total medial meniscal tear/trans-ection (MMT) through medial collateral ligament (MCL) and medial meniscus leads to abnormal joint load	1. More severe than ACLT, causing rapid degeneration 2. Affects reproducibility if the extent of injury varies slightly 3. Canines most widely used	Bendele (2001), Piskin et al. (2007), Knights et al. (2012), Kahn et al. (2016), Ali et al. (2018), Brederson et al. (2018), Pucha et al. (2020), Temp et al. (2020)
	Destabilization of medial meniscus (DMM)	Mouse Rat Rabbit	Sectioning of medial meniscotibial ligament causes DMM and load changes	1. Less severe than ACLT/slower progression 2. Easier to perform than MMT on small animals (e.g., mouse)	Glasson et al. (2007), Iijima et al. (2014), Goetz et al. (2017)
	Ovariectomy	Mouse Rat Rabbit Guinea pig Ovine	Estrogen reduction leads to accelerated cartilage erosion	1. Effect of estrogen deficiency on progression and therapeutic intervention 2. Not recommended in therapeutic trials 3. Low surgical technical difficulty so highly reproducible	Hoegh-Andersen et al. (2004), Cake et al. (2005), Dai et al. (2006), Oestergaard et al. (2006), Castaneda et al. (2008), Castaneda et al. (2010), Sniekers et al. (2010), Qin et al. (2013), Kreipke et al. (2014), Xu et al. (2019)
	Chondral/osteochondral defects	Mouse Rabbit Canine Bovine	Focal lesion by arth-rotomy triggers local inflammation and changes chondrocyte metabolism and local biomechanics	1. Study local cartilage repair and healing with therapeutics 2. Used with biomaterials in bioengineering/regenerative medicine 3. High degree of precision is required and less reproducible	Mastbergen et al. (2006), Flanigan et al. (2010), McNulty et al. (2012), Figueroa et al. (2014), Serra et al. (2014)
	Tibial osteotomy (TO)	Rat Guinea pig Rabbit Canine	Surgically-set tibial varus (or valgus) malalignment to increase mechanical loading on medial (or lateral) joint compartment	1. Biomechanical via extra-articular surgery, without any internal joint damage 2. More aligned with primary overloading OA, dependent on magnitude of malalignment 3. High surgical difficulty	Reimann (1973), Mankin et al. (1981), Johnson and Poole (1988), Panula et al. (1997), Wei et al. (1998), Britzman et al. (2018)
Non-invasive (single-impact or repetitive loading)	Intra-articular fracture of tibial plateau	Mouse Rat Rabbit Canine	Fix knee (90°) and apply impact with indenter to cause a closed fracture in the articular surface of animal lower limb	Study articular cartilage degeneration after higher-energy impact trauma injuries (e.g., frontal vehicle collisions)	Lahm et al. (2005), Rundell et al. (2005), Lewis et al. (2011), Schenker et al. (2014), Kimmerling et al. (2015), Allen et al. (2020)
	ACL rupture via tibial compression overload	Mouse Rat	Axial load to calf, fixed between upper/lower cups, leads to cranial displacement of tibia (rel. femur), overloading ACL to cause rupture	1. Rapidly developing. Similar mechanism to sports injury 2. Needs greater force (12N) applied (short time) compared to cyclic compression loading model	Lockwood et al. (2014), Khorasani et al. (2015), Brown et al. (2020)
	Cyclic articular cartilage tibial compression	Mouse Rat	Cyclic axial compressive force (4.5–9N). Setup akin to tibial compression (above), simulating overloading of ankle and knee joints	1. Study cartilage degeneration induced by chronic overload 2. Adjustable, with peak load below ACL-rupture threshold 3. Not representative of biome-chanical load environment due to muscle contractions and gait	Poulet et al. (2011), Christiansen et al. (2012), Maerz et al. (2015), Melville et al. (2015)
	Knee immobilization (Videmen model)	Rabbit	Knee was immobilized in an extended position using a custom splint applied dorsally from the thigh to the distal end of the limb	1. Periodic (e.g., 4 + 7 days cycles) or continuous immobilization models can study different chronic overload patterns 2. Immobilization periods >30 days induces progressive OA changes 3. May not fully represent human joint mechanics	Videman (1982)
	Exercise model	Mouse Rat	Trained rodents run on treadmill/wheels	1. Compatible for genetic/specific rodents to shorten process or create more severe OA. 2. May represent OA linked to occupational loading and sports	Lapvetelainen et al. (1995), Lapvetelainen et al. (2002)

The Dunkin Hartley guinea pig is the most used spontaneous model to develop naturally occurring, age-related OA. This has a high incidence and occurs at a reasonably young age compared to humans (Jimenez et al., 1997). It has similar histopathology to the human disease and the histological changes can normally be observed at 3 months of age (Kraus et al., 2010). The *Str/ort* mouse, derived from selective breeding with susceptibility to OA, is a well-recognized spontaneous OA model where >85% of all male mice have very mild OA lesions in the medial tibial plateau from 10 weeks of age (Mason et al., 2001; Staines et al., 2017a). Similarly, OA can arise spontaneously in rabbits, where natural disease can be found in >50% by the sixth year of age (Arzi et al., 2012). Spontaneous dog OA has also been proposed as a model, as OA can have high prevalence (>20%) over 1-year of age, and dogs have more human-like anatomy and OA heterogeneity than rodents (Meeson et al., 2019a). Genetically modified models, mainly in mice, are designed to knock down, knockout, knock-in or mutate specific genes to breed strains with modified OA susceptibility (Little and Hunter, 2013).

The induced methods aim to generate joint destabilization and increase joint contact force, and/or create an intra-articular inflammation, and so consequently alter cell metabolism and induce an OA lesion (Teeple et al., 2013). Chemically induced models mostly include intra-articular injection of an agent to trigger acute local inflammation, extracellular matrix degradation and chondrocyte death (Bendele, 2001). The chemical agents generally used in induced animal models of ‘OA-like’ joint pain are papain (Sukur et al., 2016), quinolone (Rodrigues-Neto et al., 2016), collagenase (Lee et al., 2020), carrageenan (Korotkyi et al., 2019), Freund’s adjuvant (Robin, 2017) and, most commonly, sodium mono-iodoacetate (MIA) (Adeyemi and Olayaki, 2017). Surgically induced models mainly use intra-articular invasive surgery to generate joint instability or create chondral defects to induce OA. PTOA is the most widely studied secondary OA model, commonly used to analyse mechanical aspects of OA. Currently, the most often used surgical models are destabilization of the medial meniscus (Arzi et al., 2012)), anterior cruciate ligament transection (ACLT; (Lampropoulou-Adamidou et al., 2014), collateral ligament transection (Bendele, 2001) other meniscal injury (meniscectomy (Ali et al., 2018) or medial meniscal tear/transection (Brederson et al., 2018; Pucha et al., 2020; Temp et al., 2020)), and chondral/osteochondral defects (Mastbergen et al., 2006; Flanigan et al., 2010; McNulty et al., 2012; Figueroa et al., 2014; Serra et al., 2014), or ovariectomy (oestrogen deficiency causing subchondral osteoblast changes) (Xu et al., 2019). Additionally, a novel non-articular invasive method was raised again recently, using tibial osteotomy (TO) to induce a varus tibial malalignment and joint mechanical overload (Britzman et al., 2018); this is more representative of the human primary chronic overloading OA condition, or a means of replicating the susceptibility to OA based on anatomical deviation, without any intra-articular damage or destabilization of internal joint mechanics (Britzman et al., 2018).

Although surgical models better mimic the pathogenic mechanisms than chemical models and have a shorter progression period than spontaneous models, they involve aseptic procedures, and the results are highly dependent on surgical skill. This means that ensuring repeatability and minimising variability of these invasive models is difficult. To address these issues, some studies create PTOA by using non-invasive methods through applying an external mechanical load to the relevant joint without disrupting the skin or the joint capsule (Christiansen et al., 2015) through either single-impact injury or repetitive loading (Poulet, 2016). There are five main types of non-surgically induced model: 1) intra-articular tibial plateau fracture (Lewis et al., 2011; Schenker et al., 2014; Kimmerling et al., 2015); 2) ACL rupture through tibial compression overload (Lockwood et al., 2014; Khorasani et al., 2015); 3) knee immobilization in an extended position (Videman, 1982) 4) cyclic articular cartilage tibial compression (Poulet et al., 2011; Melville et al., 2015); and 5) moderate running exercise. Compared to severe surgical PTOA models, these models are more likely to represent real biomechanical conditions in OA development.

The OA model classifications previously mentioned (spontaneous and induced) are rooted in the conventional view (primary and secondary OA) that distinguishes various aetiologies (intervention methods) (Table 1) (Esdaille et al., 2022). Notably, most pharmacotherapies derived from these animal models are symptomatic, primarily addressing the clinical phase of the disease rather than the pre-arthritis phase. A recent review article analyzed gene expression data across different OA models, emphasizing how discrepancies between models can lead to divergent conclusions regarding targeted intervention therapies (Soul et al., 2020). Therefore, it is crucial to clarify the commonalities among these models and understand their relationship to each etiopathogenesis and the six distinct OA clinical classifications currently adopted. A recent systematic review defined these clinical phenotypes as those related to: 1. chronic pain, 2. inflammation, 3. metabolic syndrome, 4. bone and cartilage homeostasis, 5. mechanical overload, and 6. minor joint disease phenotypes (DellIsola et al., 2016). This section summarises how these animal models relate to the clinical classifications.

Types 1 and 2. Responses to chronic pain and inflammation are usually conducted using chemically induced models, which induce cartilage degeneration based on triggering the inflammatory mechanism rapidly (Serra and Soler, 2019). The MIA method induces widespread cell death across many joint tissues leading to intense pain and is typically used to study ‘joint’ pain (Pitcher et al., 2016). These models should not be used to study the pathogenic mechanism of OA in humans (Vincent et al., 2012).

Type 3. Metabolic OA phenotype is usually characterized by surgical (ovariectomy) or spontaneous OA models. These models are appropriate to study diabetes, obesity and other metabolic disturbances (estrogen imbalance) causing OA (Xu et al., 2019).

Type 4. Bone and cartilage homeostasis phenotype presents an OA subgroup with significant changes in cartilage and bone metabolism (DellIsola et al., 2016), categorised to atrophic (few osteophytes with severe joint space narrowing) and hypertrophic (large osteophytes with little joint space narrowing) subsets (Roemer et al., 2012). The ACLT surgical model is usually used to study this phenotype, but the cyclic articular cartilage tibial compression models are also used. Bone osteophytes can be rapidly induced in experimental canine OA as early as a few days after ACL surgical induction (Gilbertson, 1975), and the subchondral and trabecular bone remodeling can be observed at around 2 months (Lavigne et al., 2005).

Type 5. The mechanical overloading phenotype which accounts for up to 22% of incidence (DellIsola et al., 2016), is usually modelled by surgical disruption of joint biomechanics, such as meniscectomy or ligament transection, causing joint instability. These models can simulate a human injury event: so-called PTOA models (Little and Hunter, 2013). However, as shown in a population study, PTOA comprises only approximately 12% of symptomatic OA (Brown et al., 2006). Therefore, it could be contended that there is an overuse of the PTOA models in representing the chronic overloading OA phenotype.

Type 6. Ageing or minor joint disease phenotypes are best studied by naturally occurring models (Lampropoulou-Adamidou et al., 2014).

The mechanical overloading phenotype itself has been shown to arise from 1) long-term overuse or cyclic small loads or 2) a short-term high load (impact) (Gardiner et al., 2016) and these two are suggested to have distinct pathophysiology progression (Little and Hunter, 2013). For example, a comparative study developed in guinea pigs with or without ACLT surgical intervention showed that there are several differences in expression of OA biomarkers between these two mechanical overload phenotypes (Wei et al., 2010; Rice et al., 2020). A later study characterised the subgroups of the mechanical overload phenotype into two: one having severe medial OA and varus malalignment with a high prevalence of injury history (55%), and the other having evidence of lateral OA and valgus alignment with lower body mass index (BMI) (DellIsola et al., 2016). Therefore, current PTOA models only represent the short impact (injury-history) subgroup, and there should be another type of biomechanically induced OA model which occurs in a relatively idiopathic, non-traumatic and slow-progressive condition to present the chronic overloading OA that is considered responsible for primary OA (DellIsola et al., 2016). The studies more representative of this form of chronic mechanical overloading OA are the non-invasive cyclic overloading model (Ko et al., 2013) and the extra-articular TO surgical model (Britzman et al., 2018). These will be described in more detail in section 4.2.

As the PTOA surgical model cannot cover all the mechanical overloading phenotypes, here, we define a new term, biomechanically induced animal models (biomechanical model for short) to present all the mechanical overloading phenotypes of OA, including those that mimic ‘primary’ mechanically induced slow-progressive OA associated with chronic overloading which is naturally occurring after long-term lower levels of increased loading (mechanical overuse), and ‘secondary’ PTOA that develops in response to acute high impact overloading or trauma. Unlike genetically modified or chemically induced models, which primarily focus on altering genetic expressions or chemical interactions within the joint, biomechanically induced models specifically recreate the mechanical conditions contributing to OA development. The main underpinning rationale is to alter the joint mechanical challenges based on changing the loading distribution, or creating joint instability or joint defects. The following section will focus on these biomechanical aspects identified as key in animal models of biomechanical mechanisms in OA development.

## 4 Biomechanical aspects of *in vivo* models of OA

This section summarises recent advances in the development of mechanical overloading models. It also highlights that there remains a gap in ensuring that these mimic with sufficient precision the clinical condition in terms of the biomechanical aspects. We hypothesise that there are two key considerations when studying mechanically-driven OA, which are: 1) how to develop a mechanical model without inflammatory sequelae, and 2) how to induce OA without significant and immediate experimental trauma. Appropriate tackling of these concerns will enable the discrimination of changes indicative of early-stage OA in both the presence and absence of such potential injury-related onset, enabling comprehensive identification of mechanisms underpinning slow-progressive disease.

### 4.1 Isolating biomechanical changes from inflammation in post-traumatic OA models

Except for some specific metabolic phenotypes (e.g., obesity) in which a systemic inflammatory state promotes local joint inflammation by adipose tissue releasing adipokines and other proinflammatory cytokines into the blood stream (Trayhurn and Wood, 2004; Berenbaum et al., 2013) to cause OA, most OA aetiology does not have a primarily inflammatory focus. The inflammation observed in most forms of OA is indeed generally chronic, low-grade inflammation and thought to be an epiphenomenon induced by mechanical derangement (Poulet et al., 2012; Sokolove and Lepus, 2013; Robinson et al., 2016; Chow and Chin, 2020). The chronic inflammation in OA is likely a secondary process induced by cartilage damage, which can be understood as a progressive cycle of local tissue damage in an initial area, failed tissue repair, and inflammatory and innate immune response, resulting in further cartilage degeneration in the surrounding area over time (Robinson et al., 2016).

The inflammation process provoked by joint trauma has two phases: an acute posttraumatic phase lasting 2 months after the mechanical impact, with joint pain and swelling due to the intraarticular bleeding, prominent inflammation and synovial effusion (Lotz and Kraus, 2010), during which the damage progresses rapidly. Then most individuals develop into the chronic OA phase, from a long clinically asymptomatic period with constant low-grade inflammation, subtle metabolic changes and structure changes in cartilage, to a symptomatic phase (Lotz and Kraus, 2010). In addition, although OA is thought to be a local disease, lacking the large scale systemic response observed in rheumatoid arthritis, the increase of serum C-reactive protein levels associated with OA symptoms suggests that there is an accompanying chronic low-grade systemic inflammatory response in these patients (Stürmer et al., 2004; Berenbaum, 2013; Cicuttini and Wluka, 2014).

As outlined, the acute injury or long-term mechanical abnormities can cause a different inflammatory process in patients. Inflammation can potentially thus arise in biomechanical OA models from any of three sources: the tissue injury from the artefacts of surgery, the acute inflammation after mechanical injury/impact and the chronic low-grade inflammation from long-term subtle mechanical changes. It has been reported that such mechanical forces can either induce or suppress inflammatory signalling cascades through the mechano-transduction mechanism (Knapik et al., 2014), which creates uncertainty about the source of inflammatory signalling in these biomechanical OA models. Currently, a key challenge needing to be resolved in PTOA models is how to develop a purely biomechanical model excluding tissue injury and inflammation following the surgery. Since current surgical OA models can potentially cause acute inflammation due to the surgery, it is not clear the extent to which the invasive surgery caused the inflammatory response or the mechanical stress initiates the OA (Deschner et al., 2003). This could result in misleading conclusions on the possible effects of mechanical factors on local and systemic inflammation, and OA progression. Interestingly, one study observed surgical inflammation in the synovium in the first week in the MMT rat model, but this resolved by week three (Salazar-Noratto et al., 2019), suggesting that the inflammatory effect might reduce to zero if the study were to progress to later time points. A non-invasive biomechanical model would be a suitable method to avoid the risk of unnatural internal joint biomechanics and localised tissue damage-induced inflammation. Such models usually initiate OA through causing a closed insult without disrupting joint function or breaking the skin and joint capsule (Christiansen et al., 2015). The earliest non-invasive model used an indenter to apply a single impact to the knee, leading to an intra-articular fracture of the tibial plateau (Furman et al., 2007). Another traumatic, but non-invasive method is the compression model, in which the limb is positioned with upper and lower loading cups fixed to the calf, and a compressive axial load (12 N peak force) is applied to cause the anterior displacement of tibia to cause ACL overload and rupture. This model has a similar mechanism as a sports injury and can result in rapid OA development (Christiansen et al., 2012). A comparative study showed the surgical (invasive) ACLT model increased anteroposterior laxity, while biomechanical (non-invasive) ACL rupture failed to modify this parameter, supporting the view that the non-invasive model represents the OA process well. However, both models produce direct intra-articular tissue damage (ACL rupture, tibial plateau fracture).

To avoid uncertainty, the molecular markers of OA could be measured from different fluids by choosing specific biomarkers of degeneration and metabolism. The local response can be studied by obtaining biomarkers from the joint synovial fluid (SF), while systemic inflammatory states representing total body level are usually collected from serum and urine (Kyostio-Moore et al., 2011). The biomarkers analysed from SF can sensitively reflect changes related to early PTOA, while urine and serum levels can be influenced by different systemic diseases or metabolic conditions (Teeple et al., 2013). Several studies have shown the differential cytokine levels in circulation (serum) and SF (Frisbie et al., 2008; Kyostio-Moore et al., 2011). Some biomarkers are less detectable in serum than in SF, or showing no clear differentiation between healthy and diseased states, as these cytokines are primarily released and consumed locally in the environment after binding to specific receptors on local cells and triggering intracellular signals (De Groote et al., 1992; Burska et al., 2014). However, in rodent studies, blood sampling is more commonly used than SF as it is easily accessible and would not affect OA progression (Burska et al., 2014). Serum change may thus only partly reflect pathology but would be a good diagnostic biomarker and disease early screening test. Serum biomarker sensitivity may be increased by adding a mechanical stimulus (such as moderate exercise) to the basic OA model; this may correspond to a later disease stage. Research has shown that exercise-induced increases in serum COMP, such as from a 30-min walk, can predict 5-year OA disease progression (Erhart-Hledik et al., 2012; Chu et al., 2018).

### 4.2 Developing less-invasive biomechanical models to better present the chronic overload phenotype to understand the early stages of non-traumatic OA

Due to the lack of suitable models, little is known about the pathophysiology of the chronic progression of biomechanically induced OA, especially in the early stage. This is potentially distinct from current PTOA models as their rapid OA induction is too severe to replicate human OA and the speed of OA progression means that it is hard to detect OA onset. Also, subchondral bone microfractures that are markers of early-stage OA, are hard to observe in current animal models (Ramme et al., 2016). Patients with chronic overloading OA without known major injuries, and PTOA patients also differ in other ways such as muscle activation, gait pattern, knee mechanics (Robbins et al., 2019) and radiological characteristics (Swärd et al., 2010). There are therefore two points which need to be further studied in order to address these two mechanically-induced forms of OA, (PTOA and chronic overloading OA): 1. lessening the invasiveness/trauma for biomechanical models to replicate the early stage of PTOA, and 2. producing a non-traumatic method to study the chronic overloading OA phenotype.

Recent studies have characterised less invasive models with lower controlled single impact to better study the early stages of PTOA. One study investigated a dose-response relationship by arthroscopy to directly load the canine medial femoral condyle to create a controlled acute injury at multiple different loading levels; magnetic resonance imaging (MRI) and symptomatic assessments were used as outcome measures (Brimmo et al., 2016). It was found that an 18 MPa impact replicated a ‘physiological’ impact equivalent to daily activities, which is near the threshold (20 MPa) for chondrocyte apoptosis; 40 MPa caused an ‘athletic trauma’, which led to degenerative changes in animal models; and a 60 MPa impact led to a ‘severe trauma’, which is associated with PTOA changes. The lowest level successfully generated subchondral bone microfractures that closely mimic those found in patients’ knees (Brimmo et al., 2016). More severe soft tissue damage models have nuanced different severity levels: e.g., DMM is moderately severe, but less so than ACLT (Glasson et al., 2007). The DMM model sections the medial meniscotibial ligament to offload the medial meniscus; this produces mid-to moderate human OA with subchondral bone defects within 4 weeks (Glasson et al., 2007; Iijima et al., 2014; Culley et al., 2015).

Studies on the chronic overloading OA phenotype focus on developing a less-invasive model by the application of controlled, long-term slowly increased loads on the knee. These studies have utilised methods such as cyclic overloading (Ko et al., 2013), running exercise (Lapvetelainen et al., 1995; Lapvetelainen et al., 2002) and creating different angles of TO (Britzman et al., 2018). These are described, in turn, below.

The cyclic compression loading model is an advanced non-invasive method of OA induction that uses loading at a lower compressive force (below ACL-rupture threshold) with a longer duration. It is the only model that imposes long-term repetitive small adjustable elevated loads (Christiansen et al., 2015). The joint failure mechanism is the direct overload on cartilage and progresses over the time, which is distinct from the single loading model where the cartilage lesion is a secondary consequence of joint destabilization and did not worsen with time alone (Poulet, 2016). The method produces cartilage and subchondral bone lesions (indicators of early-stage OA) even with very low loads (4.5 N to 9N in rodent model; (Ko et al., 2013). This non-invasive cyclic compression model is a most promising model of the chronic OA phenotype induced through mechanical overuse and also used as an adjunct (1.0N∼2.0N) to DMM which induced a positive anabolic response after DMM injury (Holyoak et al., 2019).

Another method for non-invasively applying mechanical cyclic joint loads is through elevated exercise; this has been developed with treadmill or wheel running (Lapveteläinen et al., 1995). The level of exercise would need to be sufficient to induce or accelerate OA and has been found to increase the incidence and severity of knee cartilage damage in specific mouse strains with OA susceptibility (Lapvetelainen et al., 1995; Lapvetelainen et al., 2002; Baur et al., 2011). These exercise models could enhance spontaneous models by reducing the time taken to induce OA. In addition, as the exercise model directly represents the specific type of OA observed in patients with frequent occupational and sports activities, this model could be further developed to study the effects of activity factors on OA development. There is also a view that exercise stimulates circulating levels of anti-inflammatory cytokines to counteract the low-grade systemic inflammation in patients (Warnberg et al., 2010). Therefore, exercise is not commonly used to induce OA in isolation, but it is considered a confounder and so is assessed in order to differentiate the effects of exercise from exercise plus concurrent OA disease (Frisbie et al., 2008). Systemic effects are likely complicating factors here, as in other models.

TO is another promising biomechanical method to model chronic overload-induced OA. At present it is the only way to use extraarticular surgery to create OA in an animal model. TO has the advantage of a surgically induced method that precisely mimics the OA pathogenic mechanisms of abnormal joint load distribution and constantly small overloading. TO also provides more anatomical overloading direction compared to a cyclic loading machine, and avoids the joint damage of traditional intraarticular surgery. Clinically, osteotomy has been reported for over 2000 years (Smith et al., 2013) and is used for treating knee OA to improve pain and function (Brouwer et al., 2014) by unloading the affected knee compartment. The animal osteotomy models initiate cartilage overload by removing a wedge of bone in the proximal third of the tibia, distal to the tibial tubercle (Coventry, 1985). The transtibial valgus osteotomy model is most generally used, causing a shift in mechanical axis to the lateral knee compartment (Panula et al., 1997). This method has been used to induce OA in dogs (Johnson and Poole, 1988; Panula et al., 1997), rabbits (Reimann, 1973; Mankin et al., 1981) and guinea pigs (Wei et al., 1998). These studies have revealed various levels of degenerative change in either the overloaded compartment alone (Mankin et al., 1981) or both compartments (Reimann, 1973). However, the model is not popular and has not been well developed. The reasons for this may be the high level of surgical skill required, the cost, and the technical difficulties in keeping a stable osteotomy position during post-operative rehabilitation (Panula et al., 1997). A more recent study (Britzman et al., 2018) has created a 30° varus proximal TO model in healthy rats, which successfully induced OA as measured using biomechanical evaluations, histology and circulating-telopeptides of type II collagen (CTX-II, cartilage degradation biomarker). The optimal angles to investigate biomechanical factors in the natural course of OA are not known. Typically 30° malalignment is used in animal models as this causes OA changes within 3 months, yet clinical malalignment in patients is much smaller (Cooke et al., 2007). Animal biomechanics are significantly different from human due to quadrupedalism and anatomical differences, so it is hard to compare clinical and animal malalignment angles. Larger malalignments and incisions closer to the joint induce OA more quickly (Schipplein and Andriacchi, 1991) and even larger angles produce other non-physiological effects such as subluxation (Shoji and Insall, 1973; Wei et al., 1998).

## 5 Diagnostic tools used to identify biomechanically induced OA

There are three key diagnostic tools for biomechanically induced OA: biochemical, biomechanical and imaging markers to measure the biological, functional and structural OA changes (Andriacchi, 2012). Typically, microscopic tools (biochemical) are usually used in small animals, and macroscopic tools (biomechanical and imaging) are used in large animals and humans (Kuyinu et al., 2016). Characterizing biomechanical models and outcomes relies on measuring biomechanical factors, but biomechanical changes are very subtle in animal models, in particular in rodents due to their size and quadrupedalism, and a single mechanical factor might respond to both mechanical and inflammatory stimuli and cause multiple marker changes. This subtlety hinders early detection and characterization of this mechanically driven disease in animal models. Therefore, advanced diagnostic tools are needed for animal models in order to improve our understanding of this disease.

### 5.1 Standard measurement tools

The divergent *in vivo* OA models make it difficult to establish standard measurements. Studies that correlate the cartilage degradation, bone remodelling and biomechanics with OA development, progression and compare the outcome measures usually need a healthy group to provide baseline data, or an accepted scoring reference to compare with the diseased group. To standardise the measures and reporting techniques used in OA research, the Histological-Histochemical Grading System (HHGS) is the earliest and most often used cartilage scoring system for OA assessment in both human and animal models (Rutgers et al., 2010). The Osteoarthritis Research Society International (OARSI) scoring system was then established and has become a widely used alternative as it covers a broad range from the earlier mild OA to advanced OA; it has greater inter-rater reliability (Rutgers et al., 2010). OARSI has developed a species-specific histopathology grading system for OA animal models, mainly involving the changes in cartilage, synovial membrane and subchondral bone, based on an extensive literature review (Aigner et al., 2010). This enables comparison between different animal studies of OA, yet as the induction method between these models varies, so the robustness of the OARSI comparison is not clear.

Histopathology currently remains the gold standard for cartilage evaluation and OA in animal models. Safranin-O staining of its proteoglycan content is the classic way to visualise cartilage in order to assess any loss (Rutgers et al., 2010). For imaging markers, radiography is the classic gold standard and the most widely used way of OA imaging in clinic and research (Roemer et al., 2014; Kuyinu et al., 2016). Key structural radiographic hallmarks are joint space narrowing, osteophyte formation and subchondral sclerosis (Roos et al., 2011). Although there is a lack of sensitivity and not all joint structures can be identified, joint space narrowing detected in X-ray is still the standard in the assessment of OA progression and the efficacy of therapies (Roemer et al., 2014).Biochemical markers from joint tissue or blood samples, or elsewhere are important tools for evaluating joint tissue degeneration and remodeling in OA (Rousseau and Delmas, 2007). Specifically, markers of cartilage degradation focus on the metabolic balance between the synthesis and degradation of primary cartilage components like Type II collagen (Murphy and Lee, 2005). Key markers for increased collagen synthesis in early OA stages include Procollagen II N-terminal propeptide (PIIANP) (Shinmei et al., 1993), Cartilage Oligomeric Matrix Protein (COMP), and latexin, with Type II collagen and COMP being prominent early indicators in animal studies (Appleton et al., 2007; Legrand et al., 2017; Orhan et al., 2021). For collagen degradation, Coll2-1 and C-terminal telopeptide of type II collagen (CTX-II) are widely recognized, often serving as substitutes for histological assessments in both human and animal studies (Meulenbelt et al., 2006; Duclos et al., 2010; Legrand et al., 2017). The assessment of degradative enzymes such as matrix metalloproteinases (MMP-13 and -3) and aggrecanases (ADAMTS-4 and -5) provides insights into the cartilage tissue’s catabolic state. Inflammatory markers like interleukin-6 (IL-6), interleukin-1β (IL-1β), tumor necrosis factor-alpha (TNF-α), and Coll2-1NO2, which indicate oxidative stress, are critical for understanding inflammation and mechanical stress impacts on chondrocytes (Appleton et al., 2007; Legrand et al., 2017; Orhan et al., 2021). However, the same pattern of cytokines activity (high IL-6, TNF-α, MMP-3 expression) has also been observed in inflammatory (e.g., rheumatoid) arthritis, suggesting that these markers are an atypical presentation and may reflect inflammation rather than being disease specific (Tchetverikov et al., 2005; Burska et al., 2014).

In parallel, research on bone markers helps assess bone metabolic and turnover activities, essential for understanding subchondral bone dynamics in OA (Chapurlat and Confavreux, 2016). Markers linked to osteoblast differentiation and bone formation include bone morphogenetic proteins (BMP-2, BMP-7) (Ma et al., 2020), and elements of the receptor activator of nuclear factor kappa-Β ligand (RANKL)/RANK/osteoprotegerin (OPG) system (Walsh and Choi, 2014), along with periostin (POSTN) (Chapurlat et al., 2000), fibulin-3, and fibronectin (FN1). Markers of osteoclastogenesis and bone resorption, such as type I procollagen (PINP), Sclerostin, and WNT1 (Krishnan et al., 2006; Chapurlat and Confavreux, 2016), as well as Cathepsin K, a key catalytic enzyme involved in bone extracellular matrix degradation (Chapurlat, 2014), are also pivotal. These bone markers are emerging as potential new OA markers, offering insights into the relative differentiation of the metabolic activity of subchondral and trabecular bone compared to traditional joint structural changes from imaging (Chapurlat and Confavreux, 2016).

Despite the extensive range of markers recorded, obtaining joint tissue/fluid samples is challenging, and blood samples often lack sensitivity and specificity. As a result, no universally accepted gold standard biochemical marker exists in OA research and clinical practice. The search for specific biomarkers of biomechanically induced OA is ongoing, supported by recent advances in high-throughput methods such as transcriptomics, proteomics, and metabolomics that facilitate new discoveries in both human and animal studies. (Parra-Torres et al., 2014; de Sousa et al., 2017; Sieker et al., 2018; Zhai et al., 2018; Soul et al., 2021; Hahn et al., 2022).

### 5.2 Quantitative biomechanical markers and gait analysis

Apart from the standard tools described above, mechanical factors in biomechanically induced OA are receiving more attention because of their significant influence on joint loading with both experimental and computational tools being used. Mechanical factors are experimentally quantified by gait analysis technology, which uses motion analysis, force analysis and musculoskeletal dynamics to derive internal loading on tissues (Howard et al., 2000; Cleather and Bull, 2011; Eftaxiopoulou et al., 2014). Although gait analysis and biomechanical markers have been widely used in human studies (Hamai et al., 2009; Heiden et al., 2009; Yakhdani et al., 2010; Whittle, 2014), characterising the animal mechanical behaviour is challenging (Carmo et al., 2012; Sander et al., 2012; Sagawa et al., 2013; Olive et al., 2017). Despite these challenges, rodent gait models (Table 2) have been developed and used for rheumatoid arthritis (Berryman et al., 2009) and neuropathic pain behaviour research. Although these tools have not been widely applied in OA, they can provide a rich suite of biomechanical biomarkers (Table 2).

**TABLE 2:** Quantitative biomechanical analysis and markers in animal models.

Analysis method	Technique	Description	Key values/variables	Derived values	Ref
Spatiotemporal	DigiGait; CatWalk	Space-time changes in gait cycle Expressed as percentage of gait cycle	stride length step length step width foot splay paw-print character stance time stride time swing time stride time	Limb duty factor = stance time/stride time Gait symmetry = (right stride time-left stride time)/stride time Limb phase= (left fore foot strike time-left hind foot strike time)/stride time Gait Compensation (shift to contralateral limb to protect injured limb)	Vrinten and Hamers (2003), Allen et al. (2012), Eftaxiopoulou et al. (2014), Jacobs et al. (2014), Lakes and Allen (2016), Jacobs et al. (2017)
Kinetic (dynamic)	Fixed static force plate	The forces of motion due to physical (contact) and remote (gravity) interaction with the outside world	Ground reaction forces: 3 component forces - vertical (out of floor) - braking-propulsion (horizontal shear) - mediolateral (shear) Body segment accelerations, masses, and moments of inertia	Peak vertical/braking/propulsive force and impulse 1st peak mediolateral force, 2nd peak mediolateral time and mediolateral impulse Intersegmental forces and moments, e.g., knee flexion moment (KFM); knee adduction moment (KAM) Peak KFM/KAM impulse/KAM: KFM ratio	Vilensky et al. (1994), Howard et al. (2000), Kean et al. (2011), Allen et al. (2012), Erhart-Hledik et al. (2015), Maly et al. (2015), Jacobs et al. (2017)
Kinematic	3D Optical motion tracking systems with reflective markers (e.g., Vicon) 3D biplanar video analysis	The measurement of three degrees of freedom (DOF) of rotation motion of all the joints, as well as three DOFs of translation for some joints	toe-off/propulsion angle toe clearance angle foot clearance angle touch down angle knee flexion/abduction/adduction angle knee angle femoral rotation angle sagittal ROM angles: knee ROM, ankle ROM, hip ROM	Coupled vector angle = knee adduction angle/flexion angle Swing velocity = stride length/swing time Peak angles during stance and swing phase Angular velocity patterns (characterised by peak flexion and extension velocities in swing phase)	Vilensky et al. (1994), Pereira et al. (2006), Allen et al. (2012), Eftaxiopoulou et al. (2014), Lakes and Allen (2016), Zeng et al. (2017), Agrawal et al. (2019)
Computational MSK modelling	Rigid body dynamic analysis with muscle modelling, e.g., OpenSim	Virtually recreate the mechanical function of musculoskeletal tissue to quantify internal loads	MSK geometry Gait kinematics/kinetic data (ext. force: GRF) Body segment parameters Muscle anatomy	Individual muscle forces Individual joint contact forces	Delp et al. (2007), Johnson et al. (2008), Britzman et al. (2018)
Computational finite element modelling	Stress analysis using software, e.g., MarcMENTAT, Ansys, Abaqus	Imaging geometry. MSK modelling define boundary conditions. Quantify tissue/structural response (stress, strain, deformation	Bone geometry of knee Cartilage geometry after staining Muscle forces from msk modelling Joint contact forces from msk modelling	Localised cartilage, bone, ligament and menisci stresses and strains	McErlain et al. (2011), Das Neves Borges et al. (2014), Zanjani-Pour et al. (2020)

Knee flexion moment (KFM) and knee adduction moment (KAM) have been shown in clinical OA studies as surrogate measures of joint loading that are associated with cartilage thickness (Erhart-Hledik et al., 2015; Maly et al., 2015; Agrawal et al., 2019). KAM is more significantly correlated with severe OA, while peak KFM has a greater effect in the early-stages of OA (Erhart-Hledik et al., 2015; Zeng et al., 2017). Also, it is observed that OA knees have significantly reduced joint range of motion (ROM) (Zeng et al., 2017). Other derived measures such as KAM impulse and coupled vector angle have been shown to be associated with joint loading and structural severity of OA (Kean et al., 2011; Agrawal et al., 2019). These mechanics markers have been used in human OA gait analysis and are often applied in preclinical animal studies. For example, dynamic changes in ground reaction force (GFR) and gait asymmetry can be observed as early as 1 week post-surgery in the rat MMT model (Jacobs et al., 2017). Higher peak KAM and medial knee joint contact force and decreased peak flexion and extension velocities in the swing phase of gait have also been observed (Vilensky et al., 1994; Britzman et al., 2018). Gait compensation and gait asymmetry has been found to be a biomechanical marker correlated to the severity of cartilage lesions in a rat OA model (Allen et al., 2012), but this does not have a strong correlation with SF cytokines in the spontaneous dog OA model (Allen et al., 2019). Combining spatiotemporal, kinematic and dynamic gait analysis can measure more subtle gait changes (Eftaxiopoulou et al., 2014) and, when combined with musculoskeletal modelling (Johnson et al., 2008; Britzman et al., 2018) and computational finite element modelling, this can lead to tissue-specific analysis of local biomechanical overload. This has been applied extensively in humans (Zanjani-Pour et al., 2020) and presents potential in preclinical rodent models of chronic overload OA.

### 5.3 Advanced imaging methods and markers

Advanced non-invasive 3D imaging methods accelerate OA research as they non-destructively enable the assessment of the early state of OA to directly image cartilage and subchondral microstructure changes and synovitis. MRI is applicable for early diagnosis and prediction of OA because of its ability to visualise cartilage, subchondral bone marrow lesions (BMLs), synovium, menisci, ligament and hypertrophic chondrocytes, as well as to quantify cartilage glycosaminoglycan content (Brimmo et al., 2016; Link et al., 2017; Wang et al., 2018; Accart et al., 2022). MRI techniques have been used experimentally in large animals (swine (Unger et al., 2018)) to small rodents (McErlain et al., 2012) to characterise OA pathophysiology (Głodek et al., 2016), including the ability to obtain markers from machine learning approaches to predict cartilage lesion progression (Pedoia et al., 2017). Microscopic computed tomography (MicroCT) is another powerful 3D imaging technique for non-invasive joint structural evaluation, such as BMLs, healing and remodelling and has been used for early stage subchondral change quantification in rodents (Botter et al., 2006). Enhancements include the use of contrast agents (Stewart et al., 2013), and novel segmentation to assess mineralised joint microstructural changes as a result of OA in rats (Ramme et al., 2017). Recent research has highlighted that changes in subchondral tissues–including subchondral trabecular bone deterioration, subchondral microdamage, and subchondral plate thickness–may serve as new structural markers in the early stages of knee OA. These subchondral changes, observed with advanced MRI and CT scans, seem to come prior to histological lesions in the cartilage layer (Day et al., 2001; Hayami et al., 2006; Li et al., 2013). MRI’s capability to detect BMLs has been associated with subchondral microdamage, as evidenced by microCT scans (Felson et al., 2001), and is supported by findings in a canine model, which suggested that subchondral bone marrow oedema could be one of the earliest signs of OA (Libicher et al., 2005). This was partially validated by human studies showing that subchondral bone damage might be the most predictive of radiographic OA, compared to other early-stage features such as cartilage or meniscal damage (Roemer et al., 2015; Singh et al., 2019; Preiswerk et al., 2022).

Beyond the structural imaging techniques like microCT and MRI, molecular imaging techniques are crucial for detecting selective molecular activities that serve as imaging markers over the course of OA disease. Techniques such as PET (Positron Emission Tomography) and optical imaging offer significant insights (Lim et al., 2020). PET uses radiolabeled tracers, such as the commonly used ^18^F-Fludeoxyglucose, to detect regions of heightened metabolic activity. This can reveal regional blood flow and bone remodeling conditions in human OA (Beckers et al., 2006). In a rat ACLT OA model, the PET tracer ^18^F-fluoride showed increased uptake in the subchondral bone of weight-bearing areas of the medial femur and tibia (Umemoto et al., 2010).

Optical imaging methods like bioluminescence and fluorescence imaging utilize light-emitting probes to visualize target gene expression within tissues in live animals. For instance, the transgenic murine line (Acan-CreER-Ires-Luc) with an aggrecan (Acan) gene enhancer allows indirect monitoring of the transcriptional activity of the aggrecan gene in cartilage, as reported in a post-DMM surgery model (Cascio et al., 2014). In fluorescence optical imaging, the intra-articular injection of the far-red probe Cy5.5 conjugated to an antibody selective for reactive-oxygen-damaged type II collagen has been used *in vivo* in DMM mice models, serving as an imaging marker for monitoring cartilage degradation (Lim et al., 2015).

Photoacoustic (PA) imaging is a hybrid imaging modality based on optical and ultrasound imaging. Quantitative ultrasound imaging relies on echogenicity, where strong echoes are observed at the surfaces of the cartilage and sub-cartilage bone (Saïed et al., 2009). PA imaging uses a nano-second pulsed laser to illuminate biological tissue. The absorption coefficient of the tissues, which depends on their heat and elastic characteristics, generates different pressure waves (PA signals). These signals are then detected by an ultrasonic transducer, with stronger signals in tissues with higher vascular distribution (Hagiwara et al., 2015). Ultrasound and photoacoustic imaging can use a combined US/PA transducer to visualize the OA knee, providing complementary imaging information (Izumi et al., 2013). Additionally, cartilage-targeted biochemical PA contrast agents have been developed with sufficient PA intensity and targeted interaction with GAGs in cartilage. These agents sensitively reflect the reduced content of GAGs in the DMM mice model (Xiao et al., 2020).

## 6 Discussion

Knee OA is a multifactorial complex disease which is not yet fully understood. As a mechanically-driven or mediated disease, biomechanical factors are involved in all OA phenotypes. The aim of this review was to give a narrative description of preclinical *in vivo* models and advanced measurement tools to address these biomechanical factors in the context of clinical phenotypes. We hypothesised that there are two key considerations when studying mechanically driven OA, which are: 1) how to develop a mechanical model without inflammatory sequelae and 2) how to induce OA without significant experimental trauma and so enable the detection of changes indicative of early-stage OA in the absence of such sequelae.

We first summarised the evidence for the association between biomechanical factors with knee OA, including anatomical factors (joint morphology and limb alignment) and functional factors (knee injury, poor muscle function, reduced proprioceptive acuity, activities, and joint laxity). KOA has been categorised into two phenotypes (primary and secondary OA) and several subgroups based on aetiology and risk factors, yet they are frequently overlapping and OA in many individuals likely reflects a combination of many risk factors. Cohort case studies and meta-analyses have helped to narrow these down into six phenotypes based on the clinical characteristics (DellIsola et al., 2016). As abnormal intra-articular stress is thought to be a major determinant and key feature of OA pathology in all clinical phenotypes initiated by multiple factors, the mechanical mechanism has an overwhelming importance in OA pathophysiology and treatment (Brandt et al., 2009), suggesting that all OA might share a common final pathway linking the mechanical and biological process to cause joint lesions. Treating this single causative factor (mechanical stress) might prevent the disease process in all OA phenotypes. Thus, the focus is to identify broad biomechanical principles, but more work needs to be done to characterize the effect of single biomechanical factors on OA progression when considering the complex interaction between mechanical and biological factors.

Animal models may play a critical role in identifying biomechanical factors in OA which currently still rely on results from retrospective cohort studies or theoretical biomechanical studies. A major limitation for OA model research is that the OA resulting from different aetiologies are often lumped together within the same model despite the significant heterogeneity. As the disease characteristics of OA differ between phenotypes and the phenotype-specific treatment is needed, so animal models must take these into account.

In this review we build on recent work that classifies OA into six phenotypes, of which the mechanical overloading phenotype accounts for the highest proportion of OA incidences (DellIsola et al., 2016). We propose in this article that this overloading phenotype include distinct subcategories of the PTOA subtype that occurs after trauma and, separately, a new ‘*primary’* chronic subtype that represents mechanical overloading phenotype after long-term knee overuse without known major injuries. Recent studies that compare post-traumatic OA and non-traumatic OA would seem to support this proposal. Some cohort studies have indeed found that radiological structural changes in the medial and lateral compartments are equally distributed in PTOA patients but are primarily in the medial compartment in non-traumatic patient (Swärd et al., 2010). Also, non-traumatic OA patients have higher quadriceps and lateral hamstring electromyography, and higher knee adduction angles and moments compared to PTOA (Robbins et al., 2019).

Accordingly, *in vivo* OA models need to be subcategorised and established to explicitly represent certain clinical phenotypes. Our review classifies the main models in the literature in the context of the clinical phenotypes. The comparative analysis of OA models in Table 1 underscores differences in utility for disease studies and the fidelity with which they replicate the OA process. We found that there are advances in modelling the PTOA phenotype through, for example, low impact arthroscopic insults, non-invasive ACL rupture or DMM. These have proven successful in reproducing subchondral bone changes that indicate the early stage of OA. Although there are models with external cyclic compression, extra-articular osteotomy and running exercise, a primary chronic overloading phenotype is, however, less well-modelled. Whilst invasive models are intuitively closer to PTOA and non-invasive models may be closer to the primary chronic overloading phenotype, this direct correspondence should not be assumed. Full analysis, including measures of inflammatory and biomechanical markers needs to be conducted to test these potential assumptions. If models could be developed that minimised or even stopped an acute inflammation stage and so slowed down the OA progression, then this would enable early-stage OA to be detected and analysed. Moreover, ensuring repeatability and reliability to minimize variability for these biomechanical models (both invasive and non-invasive) remains a challenge. Although these models more accurately replicate clinical conditions and thereby guide more relevant therapeutic developments, they often lack the rigorous standardization seen in chemically-induced models, which are preferred for their speed and cost-effectiveness in preliminary drug tests but fail to accurately reflect the disease’s true pathogenic processes or the underlying joint damage. Consequently, standardization is particularly vital for the widespread adoption of biomechanical models. There is a critical need for standardized protocols to enhance reproducibility across studies and boost the translational potential of research findings from biomechanical preclinical models into different subtypes of human OA.

Furthermore, for an animal model to succeed, well-defined variables and outcomes are crucial. These could include biochemical and imaging markers, mechanical measures and histological data. Advanced genetic and proteomic data can also be used for more precise phenotyping (endotypes) of these models. Lack of reliable diagnostic tools poses another concern in OA model development. This might partly explain the current poor translation from preclinical animal studies to human practice where diagnostics are entirely reliant on the presence of symptom and radiology evidence, and regulators follow this approach. Our review found that detailed quantitative biomechanical and imaging tools are not routinely used and no studies have used all of the most advanced tools simultaneously. There use in combination with other bio(chemical) markers would enable a full understanding of OA instigation and progression. Such an integrative multiscale experimental and computational framework has been applied in some large animal and human studies, and the application of this approach to small animals would facilitate many benefits due to cost, availability and genetic manipulation.

## 7 Conclusion

This review has identified a gap in the description of clinical OA phenotypes when applied to *in vivo* studies, particularly with reference to the non-traumatic chronic overloading phenotype and so we propose that the overloading phenotype include distinct subcategories of the PTOA subtype that occurs after trauma and, separately, a new ‘primary’ chronic overloading subtype that represents mechanical overloading OA phenotype after long-term knee overuse without known major injuries. This new ‘primary’ chronic overloading subtype is less well-modelled in the literature and we recommend enhanced efforts to address this. However, although invasive models are intuitively closer to PTOA and non-invasive models may be closer to the primary chronic overloading phenotype, this direct correspondence should not be assumed. Furthermore, alignment between OA onset and progression mechanisms in this ‘primary’ chronic overloading subtype and those contributing to the OA that arises in spontaneous animal models will better define their utility in translational studies.

Advances in biochemical, biomechanical and imaging biomarkers, combined with the opportunities that such new small animal models provide would enable the better development of early diagnosis of the most prevalent form of knee OA.

## Figures and Tables

**Table:** 

**Term**	**Description**
Knee malalignment	Varus or valgus alignment between femur and tibia outside the physiological range
Biomechanical aspects	The application of mechanics to biology and physiology
Biomechanical factors of osteoarthritis (OA)	Biomechanical impairment allied with forces, moments, kinematics and stresses of tissues in and around the knee joint, which play a role in OA onset or progression
Mechanical stimuli on chondrocytes	The physiological mechanical stress on the surface of chondrocytes, that is converted into intracellular chemical signals via mechano-transduction
Biomechanically induced animal models	A category of disease models, such as OA, that apply mechanical principles (including invasive and non-invasive induction methods) to a living organism by imposing altered joint loading or mechanical instability directly to the joints to recreate the mechanical conditions contributing to OA development
Mechanical overloading phenotype of OA	One of the commonest OA clinical phenotypes caused by altered joint mechanics. Herein, it is separated into ‘primary’ slow- progressive (chronic overloading) OA which occurs over long-term with low levels of elevated loading or mechanical overuse, and “secondary” post-traumatic OA after acute high impact or trauma
Biomechanical marker	Biomechanical measures of a motor state
